# Acetaldehyde and hexanaldehyde from cultured white cells

**DOI:** 10.1186/1479-5876-7-31

**Published:** 2009-04-29

**Authors:** Hye-Won Shin, Brandon J Umber, Simone Meinardi, Szu-Yun Leu, Frank Zaldivar, Donald R Blake, Dan M Cooper

**Affiliations:** 1 Department of Biomedical Engineering, University of California, Irvine, Irvine, CA 92697, USA; 2 Department Chemistry, University of California, Irvine, Irvine, CA 92697, USA; 3 Department of Pediatrics, University of California, Irvine, Irvine, CA 92697, USA

## Abstract

**Background:**

Noninvasive detection of innate immune function such as the accumulation of neutrophils remains a challenge in many areas of clinical medicine. We hypothesized that granulocytes could generate volatile organic compounds.

**Methods:**

To begin to test this, we developed a bioreactor and analytical GC-MS system to accurately identify and quantify gases in trace concentrations (parts per billion) emitted solely from cell/media culture. A human promyelocytic leukemia cell line, HL60, frequently used to assess neutrophil function, was grown in serum-free medium.

**Results:**

HL60 cells released acetaldehyde and hexanaldehyde in a time-dependent manner. The mean ± SD concentration of acetaldehyde in the headspace above the cultured cells following 4-, 24- and 48-h incubation was 157 ± 13 ppbv, 490 ± 99 ppbv, 698 ± 87 ppbv. For hexanaldehyde these values were 1 ± 0.3 ppbv, 8 ± 2 ppbv, and 11 ± 2 ppbv. In addition, our experimental system permitted us to identify confounding trace gas contaminants such as styrene.

**Conclusion:**

This study demonstrates that human immune cells known to mimic the function of innate immune cells, like neutrophils, produce volatile gases that can be measured *in vitro *in trace amounts.

## Background

Beyond the abundant respiratory gas, carbon dioxide, living organisms produce a wide variety of volatile compounds. Gas-mediated signaling is common among plant-plant, fungus-plant, insect-plant, and bacteria-plant interactions [1-7], but far less is known about such processes in mammals. Among the more extensively studied gas mediators in mammals are nitric oxide (NO) [8-15], ammonia [16], carbonyl sulfide, ethanol/acetone, and methyl nitrate [17-19]. While the potential utility of exhaled gases as a noninvasive marker of disease and metabolism is clear, knowledge of the underlying source and determinants of exhaled gases remains limited in many cases.

One relatively poorly studied but potentially significant source of physiologically active biological gases is the circulating granulocyte. In this context, NO is illustrative of the types of problems encountered; despite evidence that NO metabolic mediators are activated in neutrophils [20-22], we are unaware of studies in which NO gas has been measured directly from neutrophils *in vitro*. Other than the gases involved directly in respiration, such as O_2 _and CO_2 _which exist naturally in high concentrations, most of the remaining gases of interest found in exhaled breath exist in concentrations so small that their accurate measurement is a challenge. A related difficulty in attempting to determine gases produced by cells in culture is the fabrication of bioreactors which can accomodate a sufficient number of cells and allow ready access to the culture medium and headspace for sampling gases. Recently, analysis of human breath exhalate and smell- based medical diagnostics have been an area of rapid development [23]. Selected ion flow tube mass spectrometry (SIFT-MS), on-fibre derivatization solid-phase microextraction (derivatization/SPME) and gas chromatography mass spectrometry (GC-MS) are commonly used techniques to quantify trace amounts of volatile organic gases obtained either in exhaled human breath [17-19,24-26], or from the headspace above lung cancer cell line culture [27].

Our group, a team including expertise in biomedical engineering, immunology, translational science, and trace gas chemistry has been successful in generating novel information about breath biomarkers relevant to diseases ranging from cystic fibrosis to diabetes [17-19], and is beginning to probe the mechanisms responsible for biological trace gases. In this study, we hypothesized that human immune cells in culture can generate detectable volatile organic compounds. HL60, a well-known promyelocytic human leukemia cell line was used as a model system in this study. The goals of the current study were twofold: 1) to develop a bioreactor suitable for collecting the headspace gas above cell/media culture; and 2) apply the techniques of trace gas analysis developed in the Blake-Rowland laboratory [28]. The cells were grown in a limited, serum free medium as well as in fetal calf serum (often used in cell culture systems) in order to identify potentially confounding effects of gases likely evolved from the more complex media. A systematic approach was also used to determine contaminant gas signals (e.g., emanating from the medium, plastic culture ware, and ambient air) from signals whose source was the cells in culture.

## Methods

### Cell Culture

The HL60 cells were grown in RPMI 1640 (Gibco Ltd., Carlsbad, California, USA) supplemented with 10% fetal bovine serum (FBS) in a 37°C incubator under 5% CO_2_. The cells were transferred to the serum free media (AIM-V, Gibco Ltd., Carlsbad, California, USA) for up to 48 hours prior to the experiment to remove any conflicting growth factors provided by the FBS. On the day of the experiment, 40 × 10^6 ^cells were added to 30 ml of fresh culture medium in Teflon vials (Nalgene, Rochester, New York, USA).

### Headspace Gas Collection Equipment and Methods

**Figure 1:**
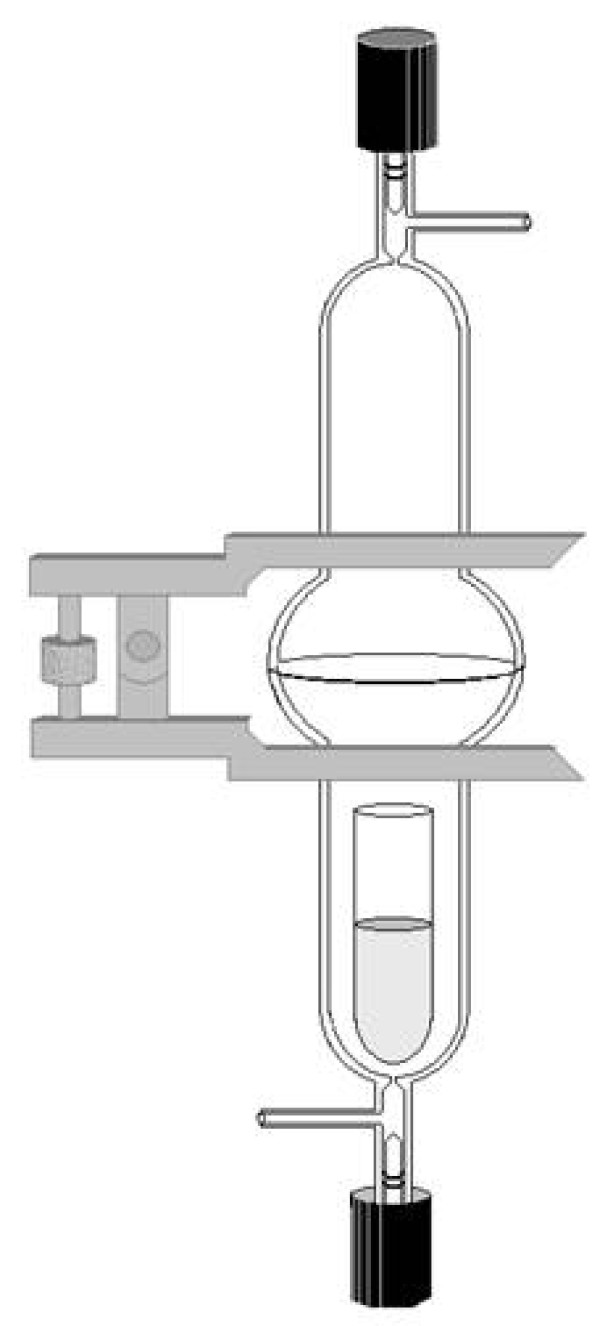
**The 378 mL glass bioreactor designed for incubating cells in air containing low volatile organic compounds and post incubation collection of the gaseous headspace**.

**Figure 2:**
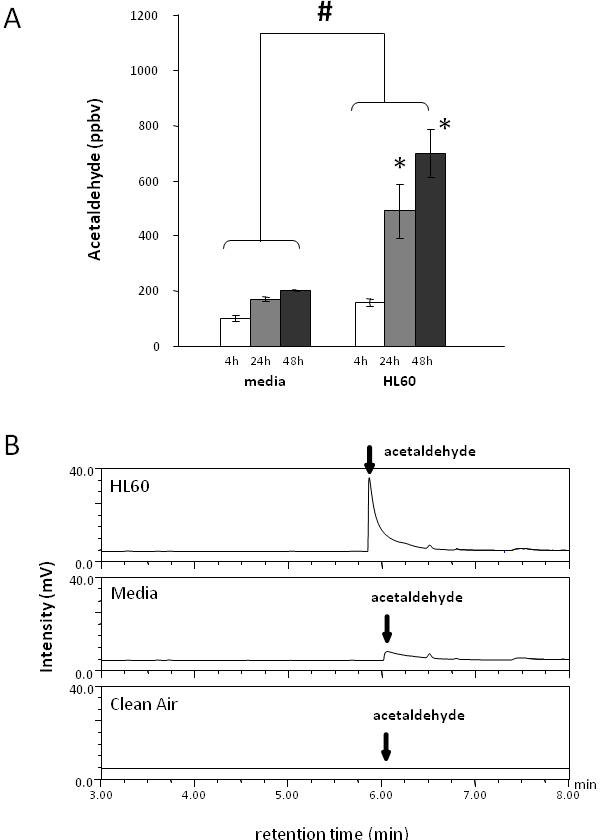
**(A) The mean ± SD acetaldehyde concentration in the bioreactor headspace of media and HL60 cells are presented at 4-h (empty bar), 24-h (gray bar) and 48-h (black bar) of incubation**. Headspace acetaldehyde concentration is significantly higher from HL60 cells compare to media (p < 0.0001). Significantly different levels of acetaldehyde are emitted at 24-h and 48-h incubations compared to 4-h from HL60 cells (4-h 157 ± 13 ppbv, 24-h 490 ± 99 ppbv and 48-h 698 ± 87 ppbv). * represents concentrations significantly higher compared to 4-h from HL60 cells, and # represents significantly higher acetaldehyde generation from HL60 cells compared to media. (B) Representative chromatograms of acetaldeyde after 48 hours of incubation. Low VOC air was used to flush the headspace of the bioreactors containing vials of media and HL60 prior to incubation.

The Teflon vials containing the cell suspensions (40 × 10^6 ^cells/30 ml) were placed inside cylindrical glass bioreactors. The glass bioreactors were specifically designed to collect the gaseous headspace above aqueous cultures (see Figure 1) [19]. The bioreactor consisted of two glass halves joined together with an o-ring and secured by a spherical joint Thomas^® ^pinch clamp. The bioreactor had an interior volume of 378 mL and was fitted with valves, sealed with high vacuum Chem-Vac™ stopcocks, at both ends. Once the apparatus was fully assembled it was attached to a pressurized manifold to purge the bioreactor of ambient air and replace it with air containing low levels of volatile organic compounds (VOCs) and 5% CO_2_. The low VOC air was prepared by doping 5% pure CO_2 _in to whole air collected by the Blake-Rowland lab from the rural Crooked Creek Research Station in California's White Mountains [29]. Figure 2(B) and 4(B) illustrate the low levels of selected VOCs in the collected air as compared to the headspace samples of the media and HL60 samples. The manifold, which was equipped with an Edwards Model vacuum pump and a 10,000 torr Edwards capacitance manometer, was capable of purging numerous bioreactors simultaneously. A needle valve (Swagelok, Solon, OH) and flowmeter (Dwyer Instruments Inc. Michigan City, Indiana, USA) was used to adjust the net flow to the bioreactors to 2500 cc/min. The purge time was adjusted, depending on the number of bioreactors in use, to ensure that each bioreactor was flushed with a volume of air approximately three times that of its own. After purging was completed, the stopcocks on each bioreactor were sealed at ambient pressure.

The bioreactors were then placed in an incubator at 37°C for the desired amount of time. After incubation, 1/4" stainless steel flex tubing was used to connect the glass bioreactor to a stainless steel canister (Swagelok, Solon, OH) [29]. The tubing was evacuated to 10^-1 ^torr and then isolated and the evacuated canister's Swagelok metal bellows valve was opened. The Teflon stopcock to the bioreactor was opened and the system was allowed to equilibrate for one minute. The canister was then closed, thereby isolating and preserving a portion of the bioreactor's headspace.

Followiong sample collection the bioreactor was disassembled and the cells were immediately collected and counted. To minimize the confounding effects of trace gases in the ambient air or from the incubated plastic culture ware, ambient room air samples were collected during purging and transfer of the bioreactor's headspace. Plastic cell culture ware and the Teflon vials were also examined as potential sources of contamination.

### Gas Chromatography-Mass Spectrometry

The analyses of the headspace gases and room samples were performed on the system previously developed by the Blake-Rowland Laboratory at UCI to measure trace atmospheric gases. A complete description of the GC parameters and analytical methods are fully discussed elsewhere [28]. Briefly, a 233 cm^3 ^(at STP) sample is cryogenically preconcentrated and injected into a multi-column/detector gas chromatography system. The system consists of three Hewlett-Packard 6890 gas chromatography (GC) units (Wilmington, Delaware, USA) with a combination of columns and detectors capable of separating and quantifying hundreds of gases, including but not limited to, nonmethane hydrocarbons (NMHC), alkyl nitrates and halocarbons in the ppm to ppq range (10^-6^–10^-15^). Nitrogen oxides, ammonia and hydrogen sulfide are not quantified with this analytical system. Preliminary identifications of the unknown signals were made using GC-MS ion fragmentation matching software (Agilent Technologies, Santa Clara, California, USA). Verification was obtained by injecting the headspace of pure compounds (diluted to ppb levels with purified UHP helium) to ensure the elution time matched that of the unknown. The mixing ratios of the oxygenates were determined using effective carbon numbers (ECN) and the linear response to carbon number of the FID, which is accurate to within 25% [30]. Concentrations of CO_2 _in the bioreactors following incubation were determined using a separate gas chromatography system. Aliquots of the collected headspace gas were injected onto a Carbosphere 80/100 packed column output to a thermal conductivity detector (TCD).

### Helium stripping

Helium stripping was used in an attempt to purge less volatile gases from the cell culture media. After 48-h incubation, the headspace above the HL60 cells and the media was collected. The Teflon vial was removed from the bioreactor and the cells were collected and counted. The supernatant was poured into a new Teflon vial and placed back into a bioreactor. The headspace of the bioreactor was then flushed for 5 minutes with purified ultra high purity (UHP) helium (Matheson, Newark, California, USA). Helium was bubbled through the media and collected in an evacuated (10^-2 ^Torr) 1.9 L stainless steel canister to a final pressure of 900 Torr. The procedure was repeated identically for the media-only condition.

### Statistics

Experiments were repeated at least three times for gas phase measurements. We applied a 2-way analysis of variance (ANOVA) to compare the gas component emitted at three incubation times (4- vs. 24- vs. 48-h) from different conditions of cell culture (media only, and HL60 cells). Data presented are mean ± standard deviation (SD) and the significance level was set at level 0.05. Multiple comparisons adjustment was applied using Bonferroni's method.

## Results

**Figure 3:**
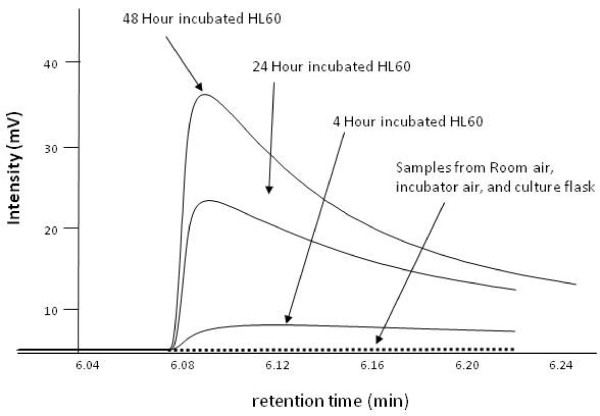
**Chromatogram of acetaldehyde from the bioreactor headspace of cells from 4-, 24- and 48-h incubations and ambient lab air**. For clarity, media chromatograms are not shown (see Fig 2 for associated media responses and standard deviations). Acetaldehyde was not present in appreciable concentrations in any of the identified sources of contamination such as Teflon vials, plastic culture ware and room air samples.

The most prominent and reproducible signal from HL60 culture was acetaldehyde. Figure 2(A) illustrates a significantly increased emission (p < 0.0001) of acetaldehyde at 24-h and 48-h compared to 4-h from HL60 cells (4-h 157 ± 13 ppbv, 24-h 490 ± 99 ppbv and 48-h 698 ± 87 ppbv), but not from the control such as media (4-h 100 ± 9 ppbv, 24-h 170 ± 8 ppbv and 48-h 202 ± 1 ppbv). The elevated acetaldehyde observed for the HL60 was significantly higher when compared with media (p < 0.0001). Figure 2(B) illustrates the insignificant levels of acetaldehyde in all other controls (i.e., room samples, empty Teflon vial, and empty culture flasks. Figure 3 is a representative chromatogram illustrating the time-dependent increase of acetaldehyde concentration in the headspace above the HL60 cells. The asymmetry of the acetaldehyde peak is a result of the oxygenate's interaction with the column, canister and manifold. Its slower desorption from the active sites of these surfaces leads to the observed tailing [30]. The asymmetry is not observed in hexanaldehyde as its behavior is dominated by its longer hydrophobic carbon tail.

**Figure 4:**
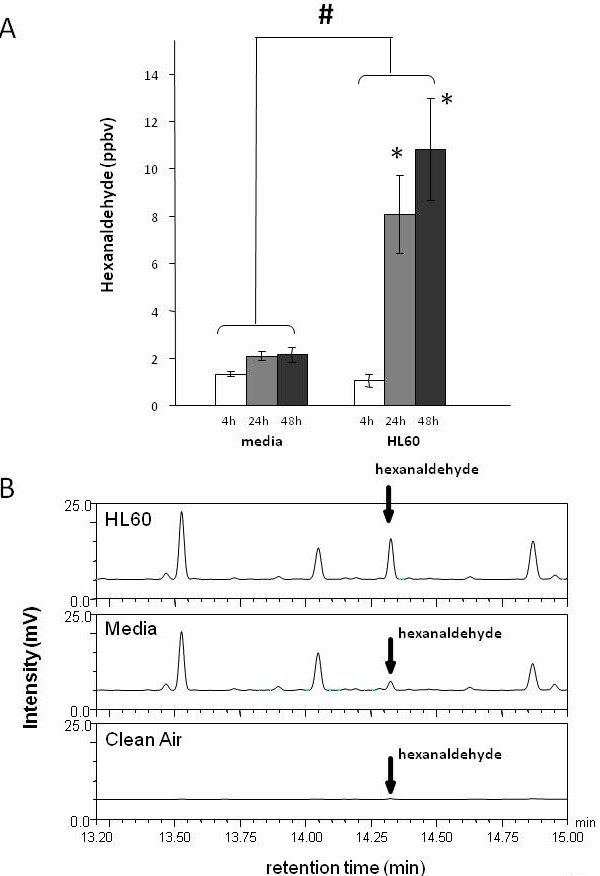
**(A) The mean ± SD hexanaldehyde concentration in the bioreactor headspace of media and HL60 cells are presented at 4-h (empty bar), 24-h (gray bar) and 48-h (black bar) of incubation**. Headspace hexanaldehyde concentration is significantly higher from HL60 cells compared to media (p < 0.0001). Significantly different levels of hexanaldehyde are emitted at 24-h and 48-h incubations compared to 4-h from HL60 cells (4-h 1.1 ± 0.3 ppbv, 24-h 8.1 ± 1.7 ppbv and 48-h 10.8 ± 2.2 ppbv). * represents concentrations significantly higher compared to 4-h from HL60 cells, and # represents significant higher hexanaldehyde generation from HL60 cells compared to media. (B) Representative chromatograms of hexanaldeyde after 48 hours of incubation. The low VOC air was used to flush the headspace of the bioreactors containing vials of media and HL60 prior to incubation. An equal volume of air was analyzed in each of the three chromatograms.

**Figure 5:**
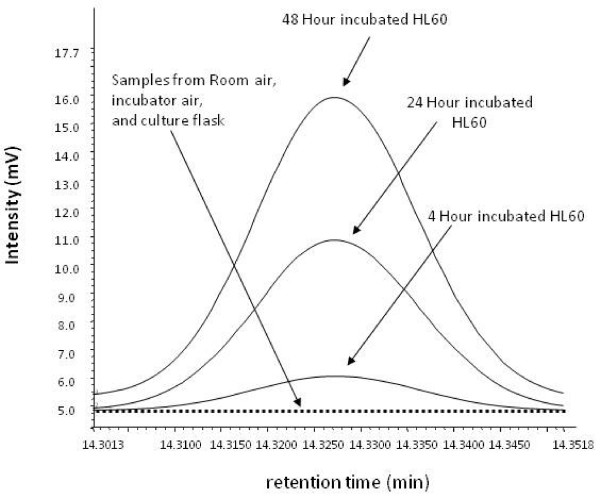
**Chromatogram of hexanaldehyde from the bioreactor headspace of HL60 cells from 4-, 24- and 48-h incubations and ambient lab air**. For clarity, media chromatograms are not shown (see Fig 4 for associated media responses and standard deviations). Hexanaldehyde was not present in appreciable concentrations in any of the identified sources of contamination such as Teflon vials, plastic culture ware, room air samples, and incubator air samples.

Hexanaldehyde was also observed to significantly increase (p < 0.0001) at 24-h and 48-h relative to 4-h in HL60 cells (4-h 1 ± 0.3 ppbv, 24-h 8 ± 2 ppbv and 48-h 11 ± 2 ppbv) but not in the media (4-h 1 ± 0.1 ppbv, 24-h 2 ± 0.2 ppbv and 48-h 2 ± 0.3 ppbv). The elevated hexanaldehyde observed for the HL60 cells was also significantly higher when compared to media (p < 0.0001) (See Figure 4(A) and 5). Hexanaldehyde was not present in appreciable concentrations in any of the identified sources of contamination such as plastic culture ware, room air samples, and incubator air samples (Figure 4(B)).

Among numerous headspace gases detected from the current HL60 study, acetaldehyde and hexanaldehyde were the only gases found in appreciable amounts from HL60 cells. In addition, no additional gases were observed when the media was stripped with helium. Although acetaldehyde and hexanaldehyde were diluted by the helium, they were still found in higher concentrations when stripped from the media in which the cells were cultured (531 ppbv and 6 ppbv, respectively) compared to the control media in which no cells were grown (126 ppbv and 2 ppbv, respectively).

HL60 cell viability decreased with incubation time. Percent survival for the HL60 cells was 93 ± 4%, 96 ± 4%, and 70 ± 6% for 4-, 24-, and 48-h incubations respectively.

**Figure 6:**
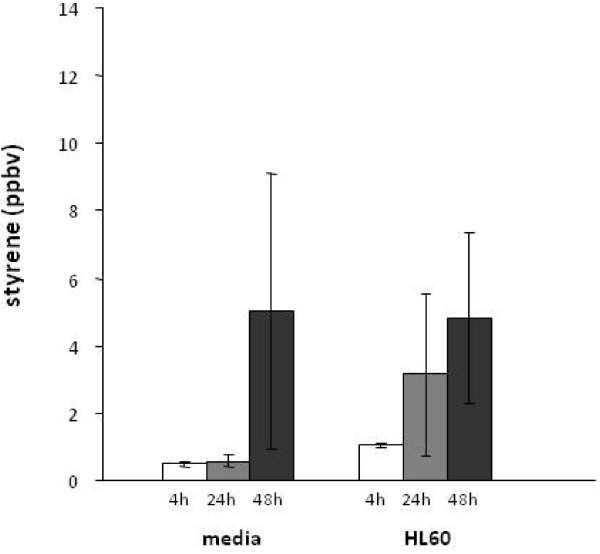
**The mean ± SD styrene concentrations in the bioreactor headspace of media and HL60 cells are presented at 4-h (empty bar), 24-h (gray bar) and 48-h (black bar) of incubation**. Styrene is an example contaminant, which originates from the cell culture flask in which the HL60 cells are grown. Styrene was seen in all the samples containing HL60 cells and media, and its responses fluctuated greatly which may be due to the various ages and exposures to the different plastic culture ware and containers in which reagents were stored.

Interestingly, several observed gas signals that increased with incubation time were later identified to be contaminants of the plastic culture ware or carry over from the fetal calf bovine serum. Styrene and 4-methyl-2-pentanone are examples of contamination. Figure 6 illustrates that styrene was seen in the samples containing HL60 cells, and media. However, the cell culture flasks in which the HL60 cells were grown were found to emit styrene. In general, styrene responses fluctuated greatly and are assumed to be due to the various ages and exposures of the different plastic culture-ware and containers in which reagents were stored (See Figure 6). A second contaminant was 4-methyl-2-pentanone. This compound was found in the ambient room air, and the headspace of media containing 10% of FBS, which was then, we believe, carried over into the samples containing cells to a significant but lesser extent. Acetaldehyde and hexanaldehyde were not observed to outgas from the plastic culture ware.

## Discussion

To the best of our knowledge, the employed trace gas characterization system, including bioreactor, and the observed acetaldehyde and hexanaldehyde from HL60 culture have not been previously reported. We found that HL60 cells generate appreciable amounts of acetaldehyde and hexanaldehyde that could be detected in the headspace above the culture media. Moreover, the experimental procedure was refined so that reproducibility of gas profiles from the cells could be observed.

Acetaldehyde has previously been detected in the exhaled human breath [31], and in human lung cancer cell line cultures [27]. The current study demonstrates that human white blood cell line, HL60 is also capable of producing acetaldehyde. When compared to the previously reported lung cancer cell line, SK-MES [27], HL60 produced similar amounts of acetaldehyde in the headspace (16-h 408 ± 191 ppbv; 24-h 490 ± 99 ppbv for 40 million of SK-MES and HL60, respectively). Until fairly recently, it was believed that acetaldehyde in human cells was produced predominately from hepatic ethanol metabolism by the enzyme alcohol dehydrogenase [32,33]. Previous studies have demonstrated that human blood cells also metabolize ethanol to acetaldehyde or oxidize it further to acetate in an alcohol dehydrogenase-independent manner [34,35]. Elegant work by Hazen and colleagues from about 10 years ago confirmed the ability of neutrophils to oxidize amino acids and produce aldehydes, a reaction requiring myeloperoxidase (MPO), hydrogen peroxide (H_2_O_2_), and chloride ion (Cl^-^) [36,37]. Since HL60 cells have high myeloperoxidase protein expression and activity [38], this amino acid oxidation is likely an alternative pathway for the generation of acetaldehyde from at least HL60 cells.

Hexanaldehyde has previously been detected in the exhaled breath [26], bronchial lavage fluid following ozone exposure [39], and exhaled breath condensate of healthy human volunteers and chronic obstructive pulmonary disease (COPD) patients [40]. Recently, elevated hexanaldehyde has been detected in whole blood from lung cancer patients compared to the healthy controls [24]. However, a cellular source of hexanaldehyde has not been completely identified. Oxidation of omega-6 unsaturated fatty acids (i.e., linoleic acid, arachidonic acid) has been reported to generate hexanaldehyde in rat and human bronchial lining fluids, and is accepted as the most plausible cellular source of hexanaldehyde [39,41-45]. As demonstrated by Babior and colleagues [46], human neutrophils are able to generate ozone as a part of their phagocyte activity. Thus, we speculate that part of the observed hexanladehyde from HL60 cells originates from the cellular reaction between cellular fatty acid and ozone.

With the exception of acetaldehyde and hexanaldehyde, all other gases quantified in the headspace of the HL60 cells were either near the detection limit of the GC-MS system, or were evolved solely from the media (i.e., pentanaldehyde). In addition, styrene was identified as a contaminant emanating from the plastic culture ware and was excluded (see Figure 6). Although the observed styrene was most likely associated with plastic culture ware, it is interesting that styrene can have biological origins [47,48].

Helium stripping is a commonly used method to detect less volatile gases dissolved in media. The purpose of helium stripping in this study was to identify gases generated by HL60 cells that would not be present in the headspace because of low volatility. However, no additional gases were observed from stripping the media with helium. This result further confirms our finding that acetaldehyde and hexanaldehyde are the major gases evolved from HL60 culture.

Over the past ten years, the interest in using exhaled gases as non-invasive markers in clinical diagnostics and therapeutic monitoring has steadily increased. In parallel, considerable efforts have been taken to understand the underlying source and determinants of exhaled volatile gases. The current study demonstrates that acetaldehyde and hexanaldehyde might be useful to identify the presence of innate immune cells like neutrophils. Moreover, these gases may also have biological importance beyond their possible role as biomarkers. For example, acetaldehyde, a known lung irritant, can influence blood coagulation [49] and induce histamine release [50-55]. The fact that these gases might be produced endogenously by neutrophils leads to the speculation that some of the deleterious effects associated, for example, with pneumonia (characterized by aggregation of neutrophils in the lung) may be due, in part, to the production of these gases by the leukocytes themselves.

## Conclusion

Our current study demonstrated a method to assess gases produced by immune cells under controlled conditions. This approach may prove useful in identifying gas "signatures" from other primary and transformed immune cell types.

## Competing interests

The authors declare that they have no competing interests.

## Authors' contributions

HWS and BJU designed and performed experiments and wrote the manuscript. SM participated in chemical analysis of volatile head space gases. SYL carried out the statistical analysis. FPZ contributed experimental design. DRB and DMC participated in the design of the experiments and provided a review of the manuscript. All authors read and approved the final manuscript.
